# Development of a novel duplex crystal digital PCR for the detection of PRRSV–1 and PRRSV–2

**DOI:** 10.3389/fcimb.2026.1701517

**Published:** 2026-02-27

**Authors:** Yuwen Shi, Jiakang He, Kaichuang Shi, Yanwen Yin, Feng Long, Shuping Feng, Zuzhang Wei

**Affiliations:** 1 Guangxi Key Laboratory of Animal Breeding, Disease Control and Prevention, College of Animal Science and Technology, Guangxi University, Nanning, China; 2 Nanning Kedi Biotechnology Co., Ltd., Nanning, China; 3 Guangxi Center for Animal Disease Control and Prevention, Nanning, China

**Keywords:** crystal digital PCR (cdPCR), differential detection, porcine reproductive and respiratory syndrome virus (PRRSV), PRRSV genotype 1(PRRSV–1), PRRSV–2

## Abstract

**Background:**

Porcine reproductive and respiratory syndrome (PRRS) is a widely prevalent disease of reproductive failure of pregnant pigs and respiratory syndromes in pigs of different ages, especially in piglets. The etiological agents include PRRS virus (PRRSV) genotypes 1 (PRRSV–1) and PRRSV–2, whereas their clinical symptoms are similar and hard to differentiate. It is necessary to establish accurate and reliable methods for differential detection of PRRSV-1 and PRRSV-2.

**Methods:**

Two pairs of specific primers and probes were designed basing on the PRRSV–1 and PRRSV–2 ORF6 gene. The reaction conditions and procedures of the duplex crystal digital PCR (cdPCR) were optimized. The specificity, sensitivity, and repeatability of the developed assay were evaluated. The application of the developed assay was assessed by testing 2,185 clinical tissue samples.

**Results:**

The results indicated that the concentration of the templates and their Ct values had good linear relationship with R^2^ of 0.998. This method could specifically detect PRRSV–1 and PRRSV–2, without cross–reaction with other swine viruses. The limits of detection (LODs) of the assay were 4.507 copies/reaction and 5.607 copies/reaction for PRRSV–1 and PRRSV–2, respectively, which was approximately 30 times more sensitive than that of the duplex real-time quantitative PCR (qPCR). The repeatability test showed that the intra– and inter–assay coefficients of variation (CVs) were 0.74%–0.93% and 0.63%–1.62%, respectively. This method was validated by testing 2,185 clinical samples from Guangxi Province in South China, and the positivity rates of PRRSV–1 and PRRSV–2 were 2.20% (48/2,185) and 23.43% (512/2,185), respectively. The coincidence rates of the developed assay with the qPCR assay recommended by the World Organisation of Animal Health (WOAH) were 99.73% and 99.73%, respectively, while with the duplex qPCR developed in this study were 99.82% and 99.77%, respectively.

**Conclusions:**

These results indicated that a rapid and accurate duplex cdPCR method with high sensitivity and excellent specificity had been successfully developed for the differential detection of PRRSV–1 and PRRSV–2.

## 1 Introduction

Porcine reproductive and respiratory syndrome virus (PRRSV) induces reproductive failure in pregnant pigs and respiratory syndromes in pigs of different ages, especially in piglets (Rossow, 1998). The main clinical signs include reproductive disorders such as miscarriage, stillbirth, mummified fetuses and weak fetuses in pregnant pigs, respiratory symptoms such as cough, runny nose, and respiratory distress in piglets, as well as fever, bleeding, and ear cyanosis in different ages of pigs (Lunney et al., 2010). PRRSV belongs to the *Arterivirus* genus in the *Arteriviridae* family, and is a single–stranded, positive–sense, and 15.5kb genome size RNA virus (Brinton et al., 2021). The RNA genome of PRRSV contains 10 open reading frames (ORFs), including ORF1a, ORF1b, ORF2a, ORF2b, ORF3, ORF4, ORF5/ORF5a, ORF6, and ORF7 (Brar et al., 2015; Kappes and Faaberg, 2015). PRRSV can be divided into two genotypes: PRRSV–1 (European type) and PRRSV–2 (North American type), with 55%–70% nucleotide identity (Shi et al., 2010a; Stadejek et al., 2013). To date, PRRSV–1 is divided into four subtypes: pan–European Subtype I, Russian Subtype I, Subtype II, and Subtype III (Shi et al., 2010a; Sun et al., 2023). Based on the ORF5 sequence, PRRSV–2 is divided into 9 lineages and 37 sublineages, while the prevalent strains in China are mainly distributed in lineages 1, 3, 5, and 8 (Shi et al., 2010b). The lineage 1 PRRSV–2 strains are the predominant strains circulating in China in recent years, which account for over 70% of cases (He et al., 2025).

The prototype of PRRSV–1, the Lelystad virus, was first isolated in the Netherlands in 1991 (Wensvoort et al., 1991); the prototype of PRRSV–2, the VR-2332 strain, was then successfully isolated in the United States in 1992 (Collins et al., 1992). In China, the first strain of PRRSV–2, CH-1a strain, was isolated in 1996 (Guo et al., 1996), and the first strain of PRRSV–1, PRRSV-FJ0602, was isolated in 2006 (Huang et al., 2008). In 2006, a new variant of PRRSV, which was more pathogenic and lethal than the original strain (Tian et al., 2007; Zhou et al., 2008), was firstly successfully isolated from pigs in Jiangxi province. The variant PRRSV was named highly pathogenic PRRSV (HP–PRRSV), and had a discontinuous deletion of 30 amino acids (aa) in nonstructural protein 2 (NSP2) (Tian et al., 2007). Since then, it has spread rapidly around the country and become the dominant prevalent strain. In 2012, the variant strain with a discontinuous deletion of 131 aa in NSP2, the NADC30–like strain, was discovered in China (Brockmeier et al., 2012; Zhou et al., 2015). In 2018, the variant strain, the NADC34–like strain, which has a continuous deletion of 100 aa (aa328–aa427) in the NSP2 region, was discovered in China (Zhang et al., 2018). At present, the strains of classical PRRSV–2, HP-PRRSV, NADC30–like and NADC34–like of PRRSV–2 are simultaneously prevalent in China (Zhou et al., 2024).

Digital PCR (dPCR) is a new generation of PCR detection technology and can be used for the detection of various pathogen nucleic acids. Firstly, the template is divided into millions of droplet reaction units. Then, PCR amplification is performed and the fluorescence signals are obtained. Finally, the number of positive and negative droplets of the sample is obtained through Poisson distribution analysis, and the copy number of the sample is counted (Pinheiro et al., 2011; Gutiérrez–Aguirre et al., 2015; Hudecova, 2015; Li et al., 2018; Tan et al., 2023). The real-time quantitative PCR (qPCR), another detection method different from the dPCR method, also is a method for real–time detection and quantification of nucleic acids (Bong et al., 2024). It can rapidly and high–throughput detect and quantify target DNA sequences in different matrices (Kralik and Ricchi, 2017). The difference between qPCR and dPCR is that dPCR is an endpoint measurement, which can accurately detect and absolutely quantify the viral nucleic acids with higher accuracy, reliability and repeatability, without relying on reference genes, standard curves, Ct values, and amplification efficiencies. It has higher sensitivity and reduced occurrence of false negative results (Kuypers and Jerome, 2017; Li et al., 2018; Tan et al., 2023). It has a relatively low sensitivity to inhibitors, which can reduce their impact on the test results (Dingle et al., 2013; Kuypers and Jerome, 2017; Demeke and Dobnik, 2018). dPCR is less affected by target sequence variability, and precision matched or exceeded that of qPCR (Hayden et al., 2013; Sedlak and Jerome, 2014; Li et al., 2018). These abovementioned advantages of dPCR make it more sensitive, accurate and reliable than qPCR, which highlights the application value of dPCR for the quantitative detection of nucleic acids, especially for the samples with low concentration of templates. At present, two distinct approaches, chamber digital PCR (cdPCR) and droplet digital PCR (ddPCR), are available to perform dPCR. The former relies on 2D arrays of microchambers to partition the sample (Ottesen et al., 2006), and the later partitions the sample in a bulk emulsion of microdroplets (Hindson et al., 2011). These two methods have been applied in biological laboratories. In this study, the Nacia system (Stilla Technologies™, Villejuif, France), with three-color multiplex capacity, was used to perform cdPCR. The Nacia system performs cdPCR combining the 2D array format of cdPCR and the droplet partitions of ddPCR (Madic et al., 2016).

Nowadays, PRRSV–1 and PRRSV–2 strains are co–circulating in different pig herds in China (Zhou et al., 2024; Qiu et al., 2025). Since they induce similar clinical signs and pathological changes, it is hard to distinguish them in terms of only clinical signs and pathological changes. To date, the commercially available vaccines of PRRSV-1 and PRRSV-2 cannot provide complete protection to different lineages within the same genotype and heterologous genotypes due to the genetic diversity [Jeong et al., 2018; Mateu et al., 2025; He et al., 2025], which further emphasizes the importance of the differential detection of PRRSV-1 and PRRSV-2 in order to use the effective vaccine strains against the prevalent strains. In addition, PRRSV–1 and PRRSV–2 have been found to co-infect pigs in some cases (Chen et al., 2019; Li et al., 2022; Huang et al., 2024; Li et al., 2024). It is necessary to accurately differentiate them depending on detection and diagnosis in laboratory in order to select the effective vaccines and adopt the correct prevention and control strategies. Currently, only PCR and qPCR have been reported for detecting and distinguishing PRRSV–1 and PRRSV–2, and no dPCR has ever been reported (Gilbert et al., 1997; Egli et al., 2001; Wasilk et al., 2004; Chae et al., 2023; Ren et al., 2025; Ruan et al., 2023; Tian X. et al., 2025). In this study, an efficient and sensitive duplex cdPCR was established for the simultaneous detection of PRRSV–1 and PRRSV–2.

## 2 Materials and methods

### 2.1 Reference strains

The vaccine strains were obtained as follows: PRRSV–2 (Ch–1R strain), porcine transmissible gastroenteritis virus (TGEV, H strain), porcine epidemic diarrhea virus (PEDV, CV777 strain), and swine influenza virus (SIV, TJ strain) from Harvac Biotechnology Co., Ltd. (Harbin, China); PRRSV–2 (R98, and JXA1–R strains), porcine pseudorabies virus (PRV, HB2000 strain) from China Animal Husbandry Industry Co., Ltd. (Chengdu, China); porcine circovirus type 2 (PCV2, ZJ/C strain), and PRRSV–2 (TJM–F92 strain) from Huapai Biotechnology Co., Ltd. (Chengdu, China); classical swine fever virus (CSFV, CVCC AV1412 strain) from Keqian Biology Co., Ltd. (Wuhan, China). The PRRSV–1 GXFS20221209 and GZ11-G1 strains were provided by our laboratory. All the strains were stored at –80 °C.

### 2.2 Clinical samples

The study protocol was approved by the Ethics Review Committee of Guangxi Center for Animal Disease Control and Prevention (CADC), China (No. 2020–A–01) on 15 November, 2020.

Between April 2024 and May 2025, 2,185 clinical tissue samples were collected from slaughterhouses, pig farms and harmless treatment plants in 14 cities of Guangxi Province, southern China. The tissues included tonsils, lymph nodes, lungs, and spleens from each pig, and the homogenized tissues from each pig were taken as one sample when tested for PRRSV. All samples were stored at –80 °C until use.

### 2.3 Design of primers and TaqMan probes

**Figure 1:**
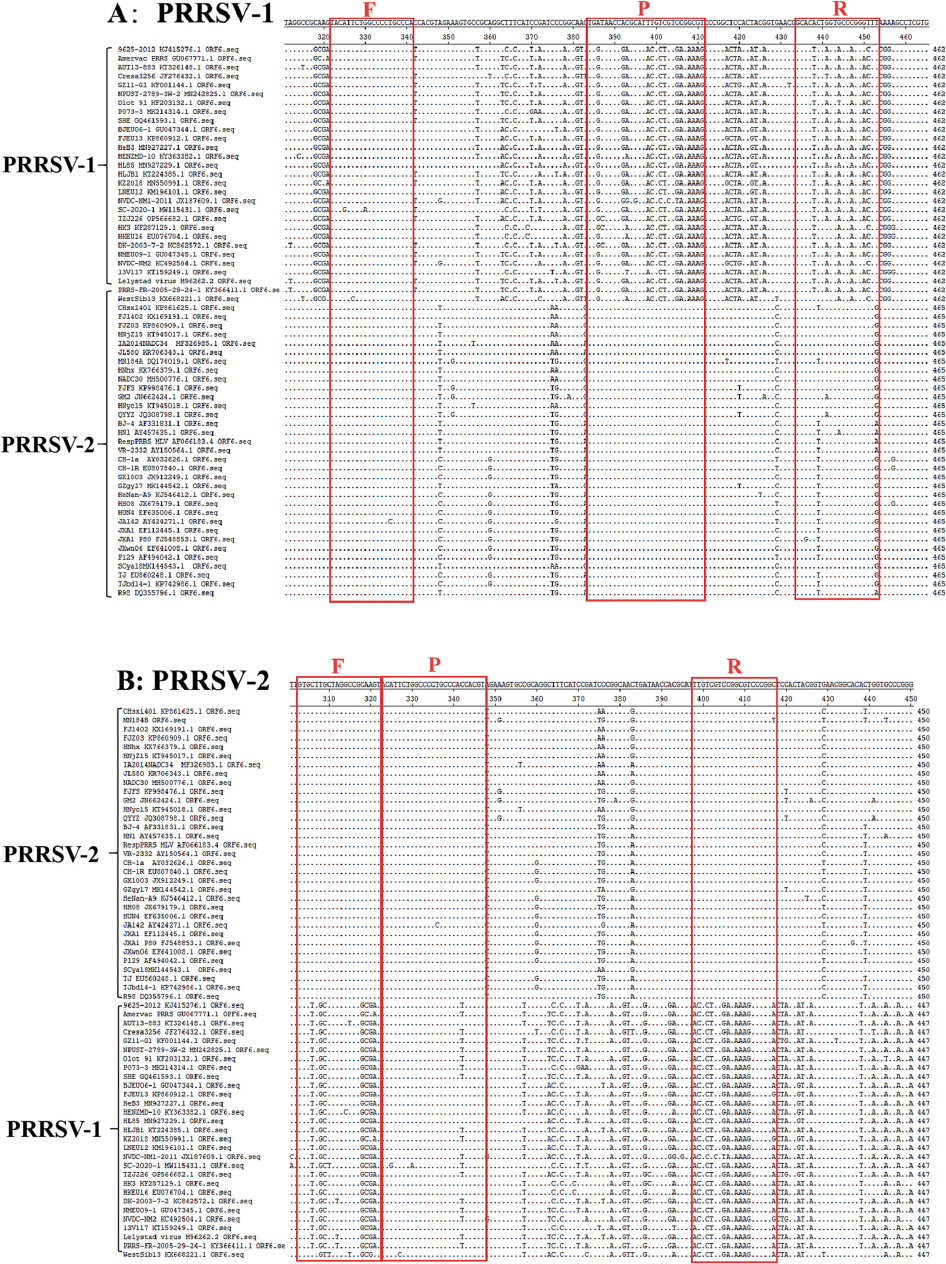
The locations of primers and probes of the duplex cdPCR. The locations of primers and probes are shown in the nucleotide sequence alignments of the PRRSV ORF6 gene **(A, B)**. F, forward primer; P, TaqMan probe; R, reverse primer.

The ORF6 gene sequences of PRRSV–1 and PRRSV–2 strains were downloaded from the NCBI GenBank (https://www.ncbi.nlm.nih.gov, accessed on 15 September 2023) (**Supplementary Table S1**). Since the global diversity of PRRSV-1 and PRRSV-2 due to the high mutation rate and recombination of PRRSV strains, these representative strains came from different countries around the world in different years in order to cover the prevalent strains of all genotypes and lineages as much as possible. The MEGA X 10.2.6 software (https://www.megasoftware.net/archived_version_active_download, accessed on 20 September 2023) was used for comparison of the ORF6 gene sequences to find the conserved regions of PRRSV (**Figure 1**). According to the conserved and differential sequences of two genotypes, the specific primers and probes were designed to distinguish the PRRSV–1 and PRRSV–2 strains using Oligo 7 software (https://www.oligo.net/doenlods.html, accessed on 25 September 2023) (**Table 1**).

**Table 1:** The designed primers and probes.

Primer/Probe	Sequence (5′→3′)	Product size (bp)
PRRSV–1–F	TACATTCTGGCCCCTGCCCA	132
PRRSV–1–R	AAGTCCTGGTACHAGAGTGC	
PRRSV–1–P	FAM–TGGTAACCGAGCATACGCTGTGAGAAAG–BHQ1	
PRRSV–2–F	GTGCTTGCTAGGCCGCAAGT	115
PRRSV–2–R	GCCGGGACGCCGGACGACAA	
PRRSV–2–F	VIC–ACATTCTGGCCCCTGCCCACCACGT–BHQ1	

The degenerated primer was designed as follows: H=A/T/C.

### 2.4 Extraction of nucleic acid

The collected clinical tissue samples were put into EP tubes, and PBS buffer (pH 7.2) (W/V=1:4) and a steel ball were added and ground for 5 minutes. Then, the samples were frozen and thawed for 3 times, centrifuged at 4 °C and 12,000 r/min for 2 min. The total nucleic acids were automatically extracted using the Nucleic Acid Extraction Kit (Zijian, Shenzhen, China) and the automatic nucleic acid extractor (Tiangen, Beijing, China) from the supernatants, and then stored at –80 °C until use.

### 2.5 Preparation of standard RNAs

The total RNAs of the PRRSV–1 GXFS20221209 strain and the PRRSV–2 CH–1R strain were extracted using the MiniBEST Viral RNA/DNA Extraction Kit Ver.5.0 (TaKaRa, Dalian, China), and reverse transcribed into cDNA. The cDNAs as templates were amplified by PCR using the specific primers (**Table 1**) containing T7 promoter, and the obtained target fragments were purified. The purified products were used as templates to transcribe RNA by T7 polymerase using the *In Vitro* Transcription T7 Kit (for siRNA Synthesis) (TaKaRa, Dalian, China). The transcribed RNAs were treated with DNase I, followed by purification using the SteadyPure Purification Kit (Accurate Biology, Hunan, China), which included precipitation, column passing, and washing steps. Finally, the purified RNAs were obtained and named sPRRSV–1 and sPRRSV–2, respectively. They were used as standard RNAs for the development and validation of the duplex cdPCR. The ultraviolet absorbance (OD_260nm_/OD_280nm_) of the standard RNAs was determined using a NanoDrop spectrophotometer (Thermo Fisher, Waltham, MA, USA), and the concentration of the standard RNAs was determined. The copy number of standard RNAs were calculated using the following formula: copy number (copies/μL) = (6.02 × 10^23^ × standard RNA concentration × 10^-9^)/(340 × standard RNA length). Then, they were diluted and stored at –80 °C.

### 2.6 Determination of reaction conditions

Firstly, a duplex qPCR for the differential detection of PRRSV–1 and PRRSV–2 was developed. The Q5 qPCR instrument (ABI, Carlsbad, CA, USA) was used to optimize the reaction conditions, including annealing temperature, primer concentration, and probe concentration. The optimal reaction conditions were determined using a total volume of 25 μL system: 12.5 μL 2× One–Step RT–PCR Buffer (TaKaRa, Dalian, China), 0.5 μL Ex Taq HS (TaKaRa, Dalian, China), 0.5 μL PrimerScript RT Enzyme Mix (TaKaRa, Dalian, China), 2.5 μL standard RNA mixture as template, two pairs of specific primers and probes (100–500 nM) with different final concentrations, and nuclease–free distilled water to a final volume of 25 μL. The duplex qPCR amplification was performed using the following procedure: 42 °C for 5 min and 95 °C for 10 s; then 40 cycles of 95 °C for 5 s, and annealing temperature (52 °C–60 °C) for 30 s.

Then, a duplex cdPCR for the differential detection of PRRSV–1 and PRRSV–2 was developed. The reaction conditions were optimized using the Naica™ sapphire crystal system (Stilla Technologies™, Villejuif, France), including annealing temperature, primer concentration, and probe concentration. The optimal reaction conditions were determined using a total volume of 25 μL system: 12.5 μL 2× PerfeCTa Multiplex qPCR ToughMix (Quanta Biosciences, Gaithersburg, MD, USA), 2.5 μL Fluorescein Sodium Salt (1μM) (Apexbio Biotechnology, Beijing, China), 2.5 μL standard RNA mixture as template, two pairs of specific primers (200–400 nM) and probes (600–1000 nM) with different final concentrations, and nuclease–free distilled water to a final volume of 25 μL. The duplex cdPCR amplification was performed using the following procedure: 45 °C for 5 min and 95 °C for 10 s; then 45 cycles of 95 °C for 5 s, and annealing temperature (55 °C–60 °C) for 30 s.

### 2.7 Generation of standard curve

The two standard RNAs sPRRSV–1 and sPRRSV–2 were mixed and serially diluted 10–fold from 1.00 × 10^8^ to 1.00 × 10^2^ copies/μL (final reaction concentration from 1.00 × 10^7^ to 1.00 × 10^1^ copies/μL). A volume of 2.5 µL of the standard RNAs was taken as template and amplified using the optimal reaction conditions of the duplex qPCR to generate the standard curves.

The two standard RNAs sPRRSV–1 and sPRRSV–2 were mixed and serially diluted 10–fold from 1.00 × 10^5^ to 1.00 × 10^1^ copies/μL (final reaction concentration from 1.00 × 10^4^ to 1.00 × 10^0^ copies/μL). A volume of 2.5 µL of the standard RNAs was taken as template and amplified using the optimal reaction conditions of the duplex cdPCR to generate the standard curves.

### 2.8 Specificity analysis

The nucleic acids of PRRSV, PEDV, TGEV, CSFV, PRV, SIV, and PCV2 were used as templates, the standard RNAs were used as positive controls, and the clinical negative sample and nuclease–free distilled water were used as negative controls. The specificity of the developed duplex qPCR and duplex cdPCR were analyzed.

### 2.9 Sensitivity analysis

The standard RNA mixture of sPRRSV–1 and sPRRSV–2 was serially diluted 10–fold from 1.00 × 10^8^ to 1.00 × 10^0^ copies/μL (final reaction concentration was 1.00 × 10^7^ to 1.00 × 10^−1^ copies/μL). The diluted standard RNAs were used as templates for amplification by the developed duplex qPCR. Furthermore, Probit regression analysis (https://www.ibm.com/cn–zh/spss, accessed on 20 February 2024) was used to evaluate the limits of detection (LODs) of the duplex qPCR. The mixture of the standard RNAs was 2–fold serially diluted as 1,000, 500, 250, 125, 62.5 copies/reaction. Each concentration was repeated 21 times during the experiment, and the number of positive amplification curves was calculated.

The standard RNA mixture of sPRRSV–1 and sPRRSV–2 was serially diluted 10–fold from 1.00 × 10^5^ to 1.00 × 10^0^ copies/μL (final reaction concentration was 1.00 × 10^4^ to 1.00 × 10^−1^ copies/μL). The diluted standard RNAs were used as templates for the duplex cdPCR method for amplification. Additionally, Probit regression analysis (https://www.ibm.com/cn–zh/spss, accessed on 20 February 2024) was used to evaluate the LODs of the duplex cdPCR. The mixture of the standard RNAs was 2–fold serially diluted as 25, 12.5, 6.25, 3.13, 1.56, 0.78, 0.39 copies/reaction. Each concentration was repeated 21 times during the experiment, and the number of positive amplification curves was calculated.

### 2.10 Repeatability analysis

The standard RNA mixture of sPRRSV–1 and sPRRSV–2 was diluted to 1.00 × 10^8^, 1.00 × 10^6^, and 1.00 × 10^4^ copies/μL (final reaction concentration was 1.00 × 10^7^, 1.00 × 10^5^, and 1.00 × 10^3^ copies/μL), respectively, and used as templates. Amplification was done using the optimal reaction conditions of the duplex qPCR. The intra– and inter–assay tests were conducted, and each concentration was repeated three times. The coefficients of variation (CVs) were calculated to evaluate the repeatability of the duplex qPCR.

The standard RNA mixture of sPRRSV–1 and sPRRSV–2 was diluted to 1.00 × 10^5^, 1.00 × 10^4^, and 1.00 × 10^3^ copies/μL (final reaction concentration was 1.00 × 10^4^, 1.00 × 10^3^, and 1.00 × 10^2^ copies/μL), respectively, and used as templates. Amplification was performed using the optimal reaction conditions of the duplex cdPCR. The intra– and inter–assay tests were conducted, and each concentration was repeated three times. The CVs were calculated to evaluate the repeatability of the duplex cdPCR.

### 2.11 Evaluation of the clinical samples

To evaluate the application of the developed duplex cdPCR, the 2,185 clinical samples from Guangxi Province during 2024–2025 were tested using the established duplex qPCR, duplex cdPCR, as well as the recommended qPCR of WOAH as a reference method (WOAH, 2021). The clinical sensitivity and specificity of the established methods were evaluated, and the consistency of three methods were determined.

To robustly assess the true sensitivity and specificity of the developed cdPCR, a total of 50 positive samples were selected randomly to amplify the ORF5 and Nsp2 gene regions and perform sequence analysis for genotyping confirmation.

## 3 Results

### 3.1 Preparation of positive standard RNAs

The total RNAs were extracted from PRRSV–1 and PRRSV–2 solution, and transcribed into cDNAs. The PRRSV ORF6 gene fragments of PRRSV–1 and PRRSV–2 were amplified by PCR using the specific primers. The PCR products were purified, transcribed to RNA *in vitro* using T7 polymerase. The obtained RNAs were purified, and named sPRRSV–1 and sPRRSV–2. Their concentrations were calculated, and used as the standard RNAs during development and validation of the duplex cdPCR. The results showed that the initial concentrations of the standard RNAs sPRRSV–1 and sPRRSV–2 were 6.04 × 10^10^ and 4.63 × 10^10^ copies/µL, respectively. They were diluted to 1.00 × 10^10^ copies/µL, and used as standard positive control for subsequent experiments.

### 3.2 Optimization of reaction conditions

The annealing temperature, primer and probe concentrations of the duplex qPCR were optimized to obtain the optimal reaction conditions. They were selected as the lowest Ct value, the highest ΔRn, the steepest exponential phase, and the stable plateau phase. Finally, the optimal reaction system (**Table 2**) and procedure were determined as follows: 42 °C for 5 min and 95 °C for 10 s; then 40 cycles of 95 °C for 5 s and 56 °C for 30 s. The sample with Ct value ≤ 36 was determined as positive.

**Table 2:** The reaction systems for the duplex qPCR and the duplex cdPCR.

Reagent	cdPCR		qPCR	
	Volume (µL)	Final concentration (nM)	Volume (µL)	Final concentration (nM)
PerfeCta Multiplex qPCR ToughMix (2×)	12.5	1×	/	/
Fluorescein Sodium Salt (1 µM)	2.5	100	/	/
2× One Step RT–PCR Buffer	/	/	12.5	/
Ex Taq HS (5 U/μL)	/	/	0.5	/
PrimerScript RT Enzyme Mix	/	/	0.5	/
PRRSV–1–F	0.9	900	0.4	400
PRRSV–1–R	0.9	900	0.4	400
PRRSV–1–P	0.4	400	0.4	400
PRRSV–2–F	0.8	800	0.3	300
PRRSV–2–R	0.8	800	0.3	300
PRRSV–2–P	0.3	300	0.3	300
Total nucleic acid	2.5	/	2.5	/
Nuclease–free distilled water	Up to 25	/	Up to 25	/

**Figure 2:**
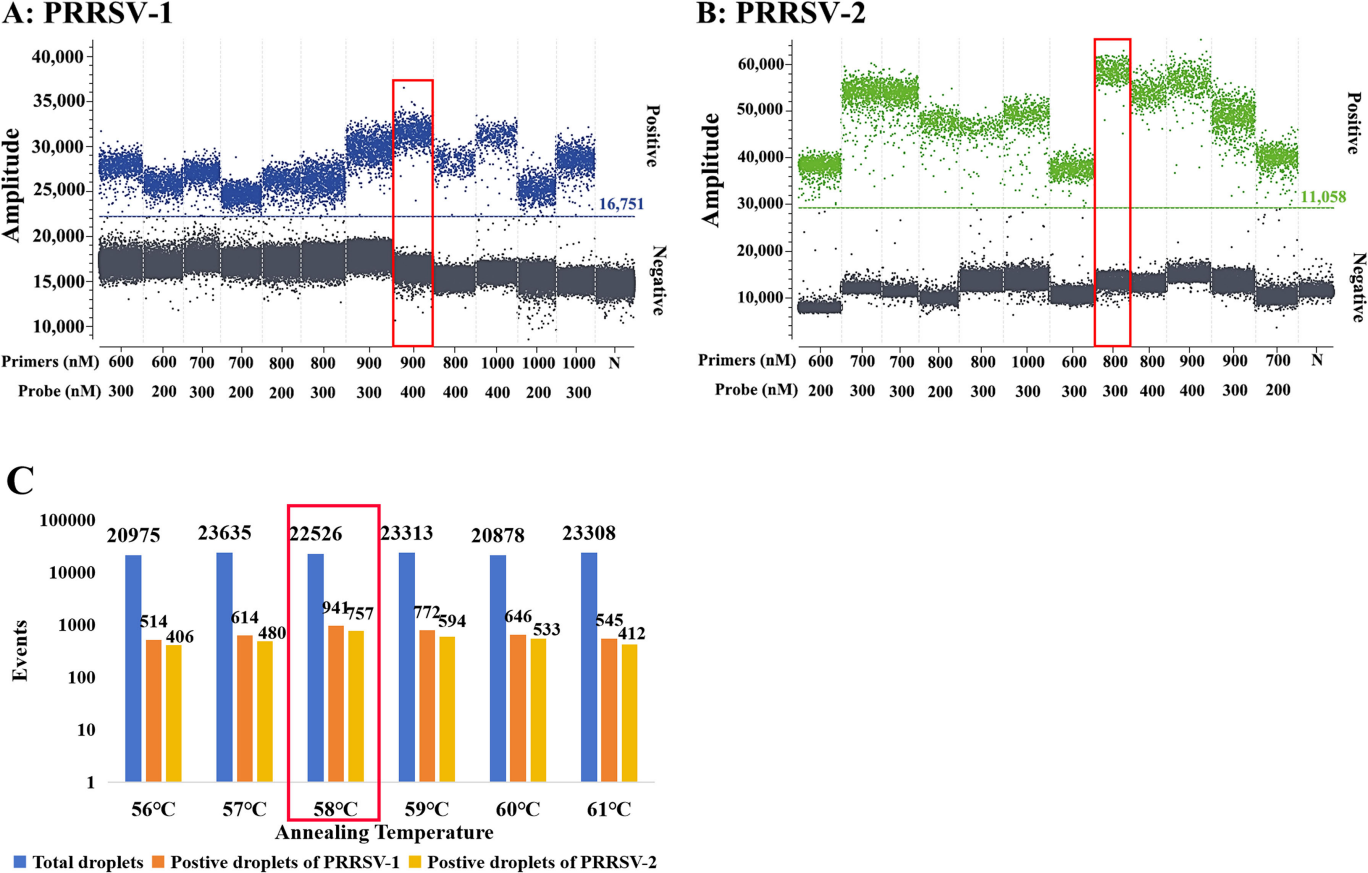
Optimization of the reaction conditions for the duplex cdPCR. **(A)** PRRSV–1; **(B)** PRRSV–2; **(C)** Annealing temperature.

The annealing temperature, primer and probe concentrations of the duplex cdPCR were optimized to obtain the optimal reaction conditions. They were determined according to a significant distinction between positive and negative droplet clusters, a small number of dispersed droplets in the middle, and the high fluorescence signal values of positive droplets. Finally, the optimal reaction system (**Table 2**) and procedure were determined as follows: 45 °C for 5 min and 95 °C for 10 s; then 45 cycles of 95 °C for 5 s and 58 °C for 30 s (**Figure 2**).

### 3.3 Generation of standard curves

**Figure 3:**
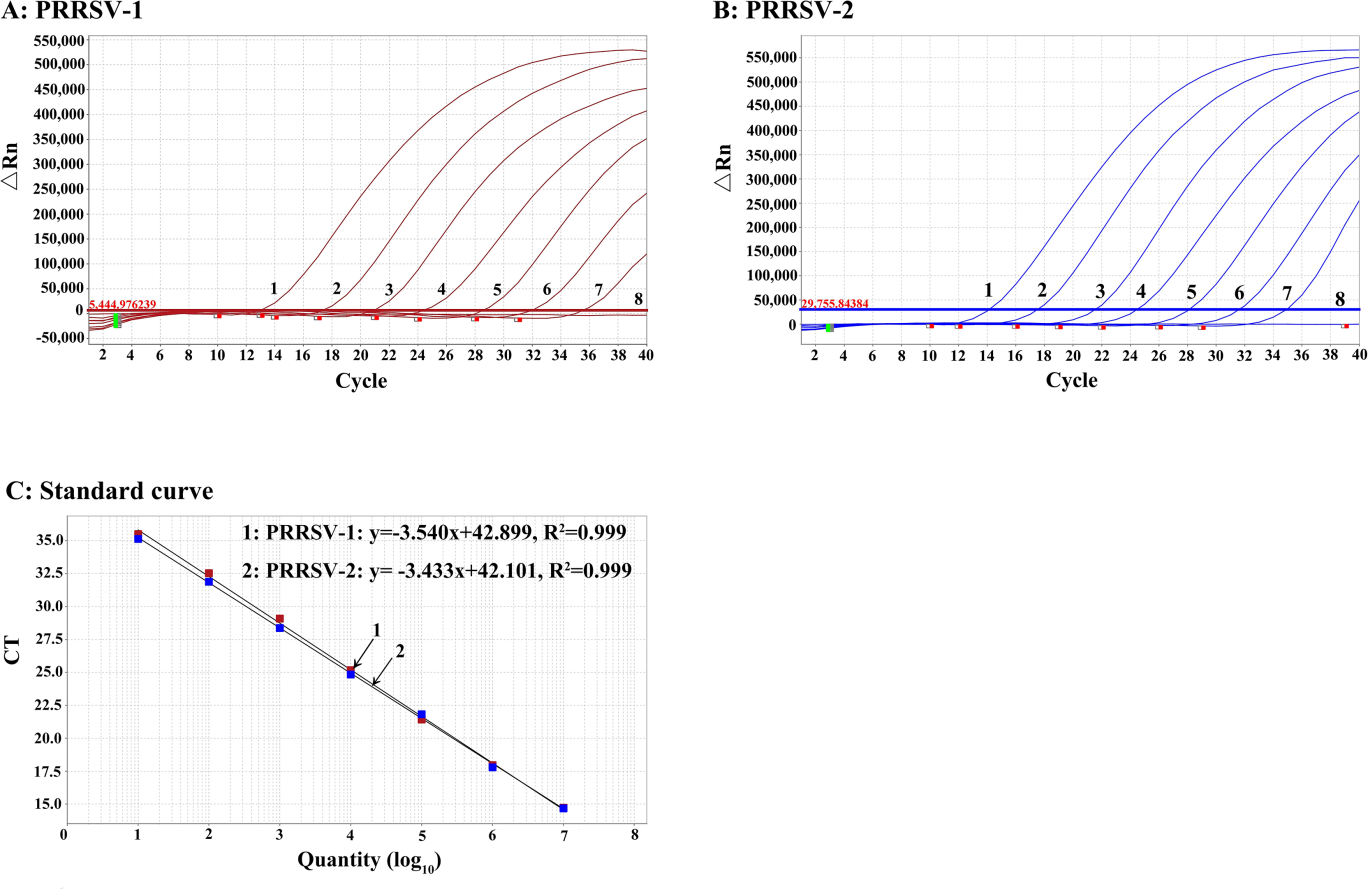
Generation of the standard curves of the duplex qPCR **(A–C)**. Amplification curves of PRRSV–1 **(A)** and PRRSV–2 **(B)**. **(C)** Standard curves. 1–7: The final reaction concentration of the standard RNAs ranged from 1.00 × 10^7^ to 1.00 × 10^1^ copies/μL; 8: Negative control.

The two standard RNAs sPRRSV–1 and sPRRSV–2 were mixed and serially diluted 10–fold from 1.00 × 10^8^ to 1.00 × 10^2^ copies/μL (final reaction concentration from 1.00 × 10^7^ to 1.00 × 10^1^ copies/μL) to generate the standard curves. The results showed that the correlation coefficient (R^2^) of PRRSV–1 and PRRSV–2 were 0.999 (**Figure 3**).

**Figure 4:**
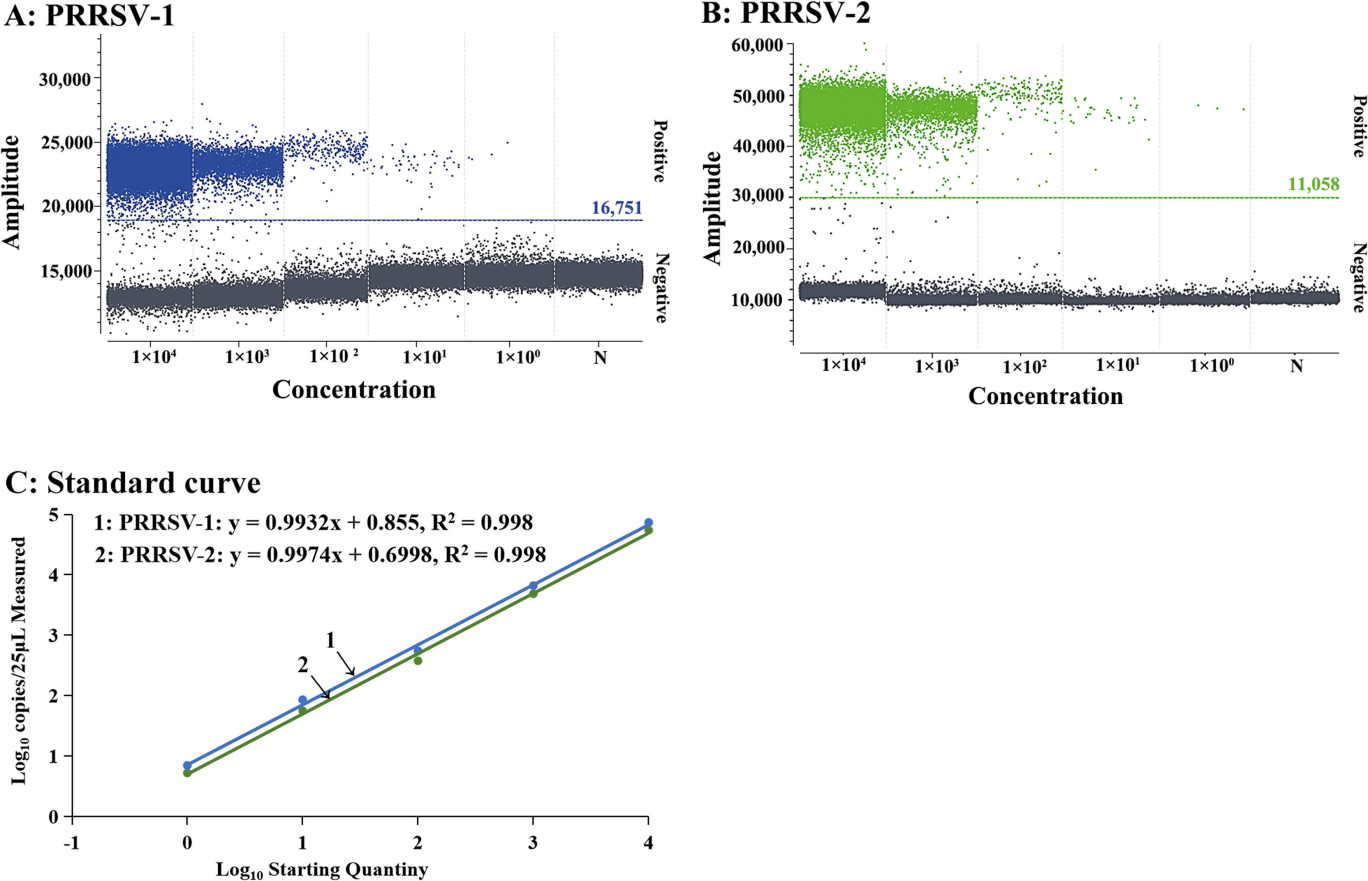
Generation of the standard curves of the duplex cdPCR. Amplification of PRRSV–1 **(A)** and PRRSV–2 **(B)** from 1.00 × 10^4^ to 1.00 × 10^0^ copies/μL (final reaction concentration). **(C)** Standard curves.

The two standard RNAs sPRRSV–1 and sPRRSV–2 were mixed and serially diluted 10–fold from 1.00 × 10^5^ to 1.00 × 10^1^ copies/μL (final reaction concentration from 1.00 × 10^4^ to 1.00 × 10^0^ copies/μL) to generate the standard curves. The results showed that the correlation coefficient (R^2^) of PRRSV–1 and PRRSV–2 were 0.998, indicating this method has a good linear relationship (**Figure 4**).

### 3.4 Specificity

**Figure 5:**
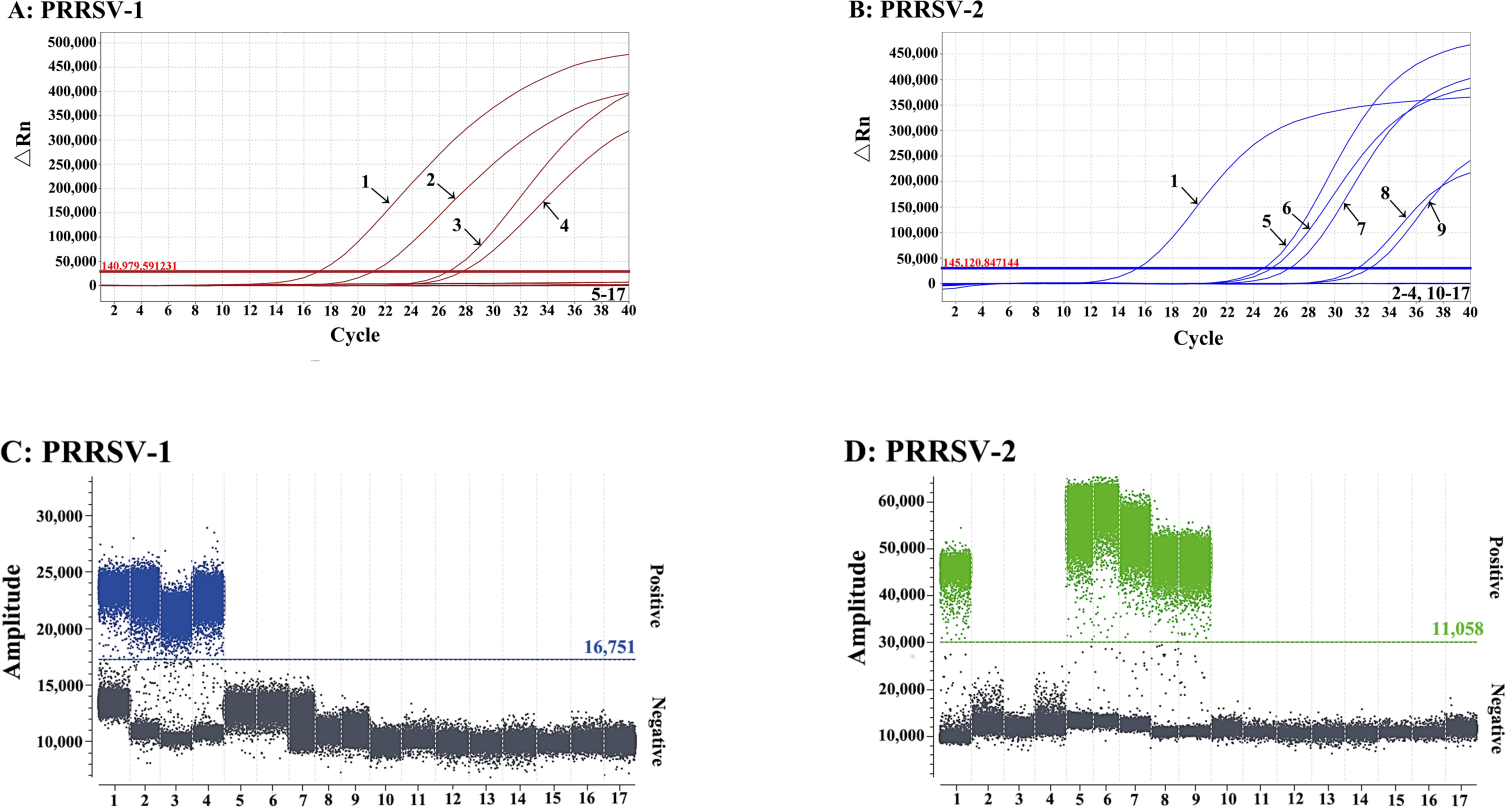
Specificity of the duplex qPCR for PRRSV–1 **(A)** and PRRSV–2 **(B)**, and of the duplex cdPCR for PRRSV–1 **(C)** and PRRSV–2 **(D)**. 1: sPRRSV–1 and sPRRSV–2 standard RNA mixture; 2: sPRRSV–1 standard RNAs; 3: PRRSV–1 GZ11–G1 strain; 4: PRRSV–1 GXFS20221029 stain; 5: sPRRSV–2 standard RNAs; 6: PRRSV–2 TJM–F92 strain; 7: PRRSV–2 CH–1R strain; 8: PRRSV–2 JXA1–R strain; 9: PRRSV–2 R98 strain; 10–15: PEDV, TGEV, CSFV, PRV, SIV, and PCV2; 16: Clinical negative sample; 17: Nuclease–free distilled water.

The nucleic acids of PRRSV, PEDV, TGEV, CSFV, PRV, SIV, and PCV2 were used as templates to analyze the specificity of the developed duplex qPCR and the developed duplex cdPCR. The results showed that two assays could specifically detect only PRRSV, and had no cross–reaction with other common porcine viruses (**Figure 5**).

### 3.5 Sensitivity

**Figure 6:**
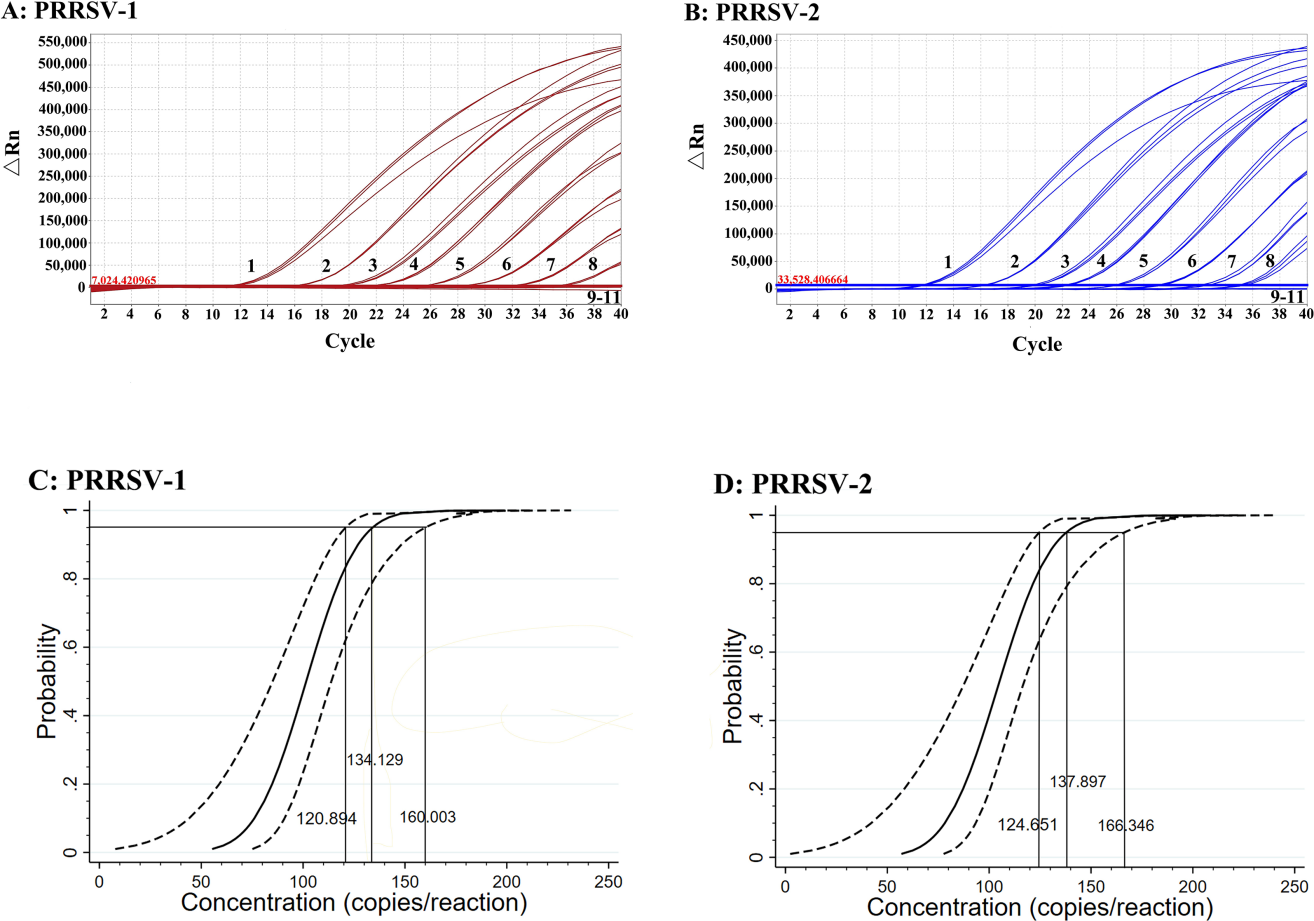
Sensitivity analysis of the duplex qPCR for PRRSV–1 **(A)** and PRRSV–2 **(B)** using 10-fold serial dilution, and sensitivity analysis of the duplex qPCR using Probit regression analysis for PRRSV–1 **(C)** and PRRSV–2 **(D)**. In A-B, 1–10: the standard RNA mixture ranged from 1.00 × 10^8^ to 1.00 × 10^−1^ copies/μL (final reaction concentration); 11: Nuclease–free distilled water.

The standard RNA mixture of sPRRSV–1 and sPRRSV–2 was serially diluted 10–fold from 1.00 × 10^8^ to 1.00 × 10^0^ copies/μL (final reaction concentration from 1.00 × 10^7^ to 1.00 × 10^-1^ copies/μL) to determine the sensitivity of the duplex qPCR. The results showed that the LODs of PRRSV–1 and PRRSV–2 was 10 copies/μL (**Figure 6**). In addition, the two standard RNA mixture of 1,000, 500, 250, 125, and 62.5 copies/reaction (final reaction concentration) were used to determine the LODs of PRRSV–1 and PRRSV–2 in the duplex qPCR by Probit regression analysis. The results are shown in **Table 3**. The LODs of PRRSV–1 and PRRSV–2 were 134.129 (95% CI: 120.894–160.003), and 137.897 (95% CI: 124.651–166.346) copies/reaction (**Figure 6**), respectively.

**Table 3:** The Ct values and hit rates of the serially diluted standard RNAs for the duplex qPCR and the duplex cdPCR.

Positive standard	Number of samples	Duplex qPCR			Duplex cdPCR		
		Concentration (Copy/reaction)	Ct (average)	Hit rate (%)	Concentration (Copy/reaction)	Positive	Hit rate (%)
sPRRSV–1	21	/	/	/	25.00	21	100.00
	21	/	/	/	12.50	21	100.00
	21	1000	31.45	100	6.25	21	100.00
	21	500	33.88	100	3.13	14	66.67
	21	250	34.54	100	1.56	8	38.10
	21	125	35.69	90.48	0.78	3	14.29
	21	62.50	ND	0	0.39	0	0
sPRRSV–2	21	/	/	/	25.00	21	100.00
	21	/	/	/	12.50	21	100.00
	21	1000	31.24	100	6.25	20	95.24
	21	500	33.62	100	3.13	13	61.90
	21	250	34.22	100	1.56	9	42.86
	21	125	35.53	85.71	0.78	3	14.29
	21	62.50	ND	0	0.39	0	0

**Figure 7:**
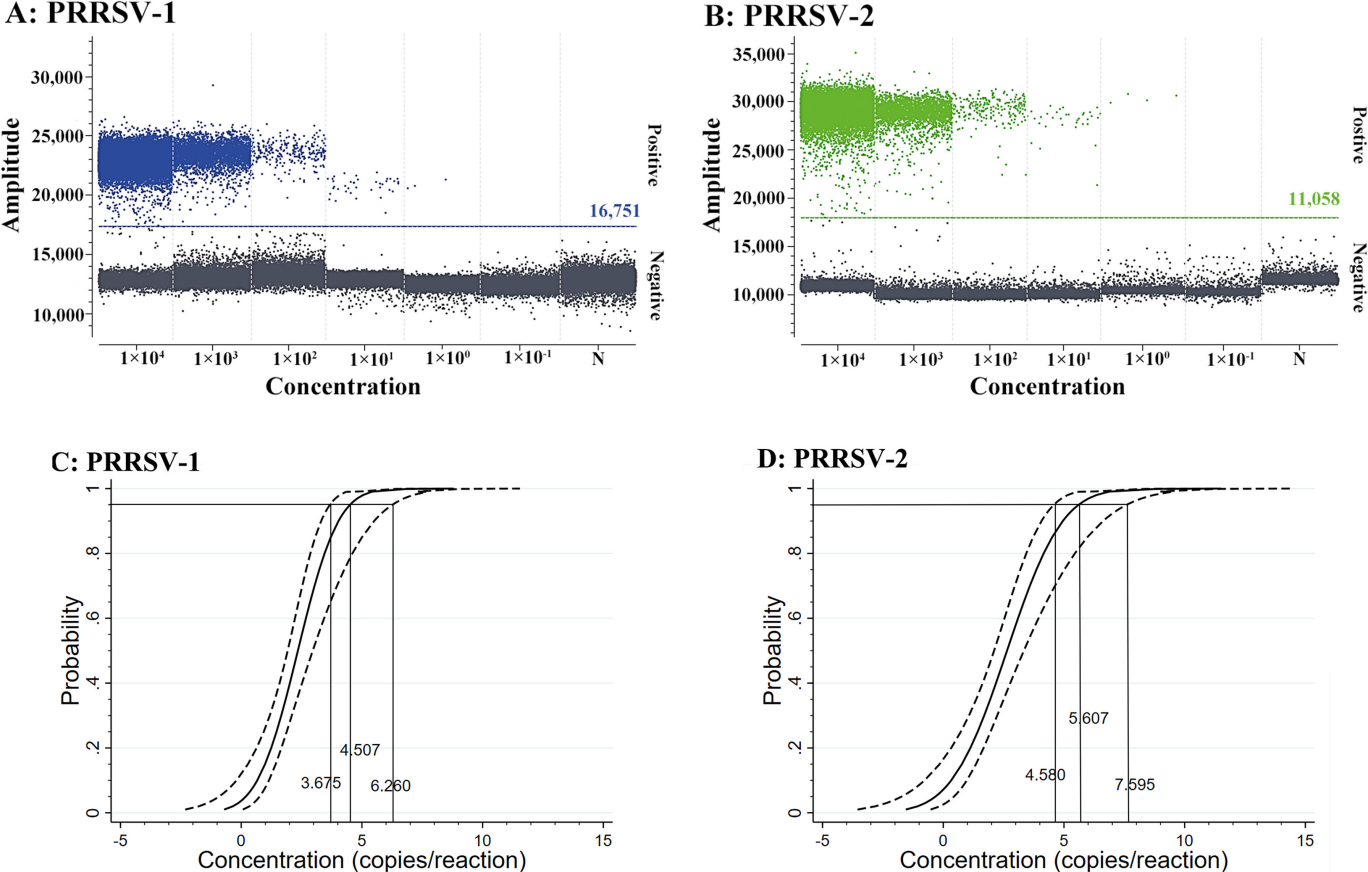
Sensitivity analysis of the duplex cdPCR for PRRSV–1 **(A)** and PRRSV–2 **(B)** using 10-fold serial dilution, and sensitivity analysis of the duplex cdPCR using Probit regression analysis for PRRSV–1 **(C)** and PRRSV–2 **(D)**. In **(A, B)**, the standard RNA mixture ranged from 1.00 × 10^4^ to 1.00 × 10^−1^ copies/μL (final reaction concentration). N: Nuclease–free distilled water.

The standard RNA mixture of sPRRSV–1 and sPRRSV–2 was serially diluted 10–fold from 1.00 × 10^5^ to 1.00 × 10^0^ copies/μL (final reaction concentration from 1.00 × 10^4^ to 1.00 × 10^−1^ copies/μL) to determine the sensitivity of the duplex cdPCR. According to the analysis results of the Poisson distribution, the LODs of PRRSV–1 and PRRSV–2 were 4.50 and 5.25 copies/μL (**Figure 7**), respectively. In addition, the two standard RNA mixture of 25, 12.5, 6.25, 3.13, 1.56, 0.78, and 0.39 copies/reaction (final reaction concentration) were used to determine the LODs of PRRSV–1 and PRRSV–2 in the duplex cdPCR by Probit regression analysis. The results are shown in **Table 3**. The LODs of PRRSV–1 and PRRSV–2 were 4.507 (95% CI: 3.675–6.260), and 5.607 (95% CI: 4.580 –7.595) copies/reaction, respectively (**Figure 7**).

### 3.6 Repeatability

The repeatability of the duplex qPCR was evaluated via three different concentrations of the standard RNA mixture as templates: 1.00 × 10^7^, 1.00 × 10^5^, and 1.00 × 10^3^ copies/μL (final reaction concentration). The results showed that the intra– and inter–assay CVs were 0.24%–1.25% and 1.11%–1.78%, respectively (**Table 4**).

**Table 4:** Repeatability analysis of the duplex qPCR and the duplex cdPCR.

Positive standard	Duplex qPCR							Duplex cdPCR						
	Concentration (Copy/μL)	Intra–assay (Ct value)			Inter–assay (Ct value)			Concentration (copy/μL)	Intra–assay (Copy/reaction)			Inter–assay (Copy/reaction)		
		$\bar{X}$	SD	CV (%)	$\bar{X}$	SD	CV (%)		$\bar{X}$	SD	CV (%)	$\bar{X}$	SD	CV (%)
sPRRSV–1	1.00 × 10^7^	12.81	0.03	0.24	13.03	0.21	1.63	1.00 × 10^4^	63,933.33	474.49	0.74	6,5601.39	1,179.495	1.18
	1.00 × 10^5^	20.58	0.26	1.25	20.45	0.36	1.78	1.00 × 10^3^	6,331.67	55.24	0.87	6,295.56	54.04	0.86
	1.00 × 10^3^	27.31	0.29	1.07	27.64	0.42	1.51	1.00 × 10^2^	478.33	4.25	0.89	480.84	3.54	0.74
sPRRSV–2	1.00 × 10^7^	13.71	0.05	0.36	13.47	0.20	1.45	1.00 × 10^4^	52,641.67	418.99	0.80	52,563.39	331.06	0.63
	1.00 × 10^5^	20.48	0.10	0.47	20.50	0.26	1.27	1.00 × 10^3^	4,808.33	44.93	0.93	4,918.33	79.77	1.62
	1.00 × 10^3^	27.14	0.30	1.10	27.53	0.31	1.11	1.00 × 10^2^	478.33	4.25	0.89	357.50	4.08	1.14

The repeatability of the duplex cdPCR was evaluated via three different concentrations of the standard RNA mixture as templates: 1.00 × 10^4^, 1.00 × 10^3^, and 1.00 × 10^2^ copies/μL (final reaction concentration). The results showed that the intra– and inter–assay CVs were 0.74%–0.93% and 0.63%–1.62%, respectively (**Table 4**).

### 3.7 Detection results of the clinical samples

**Figure 8:**
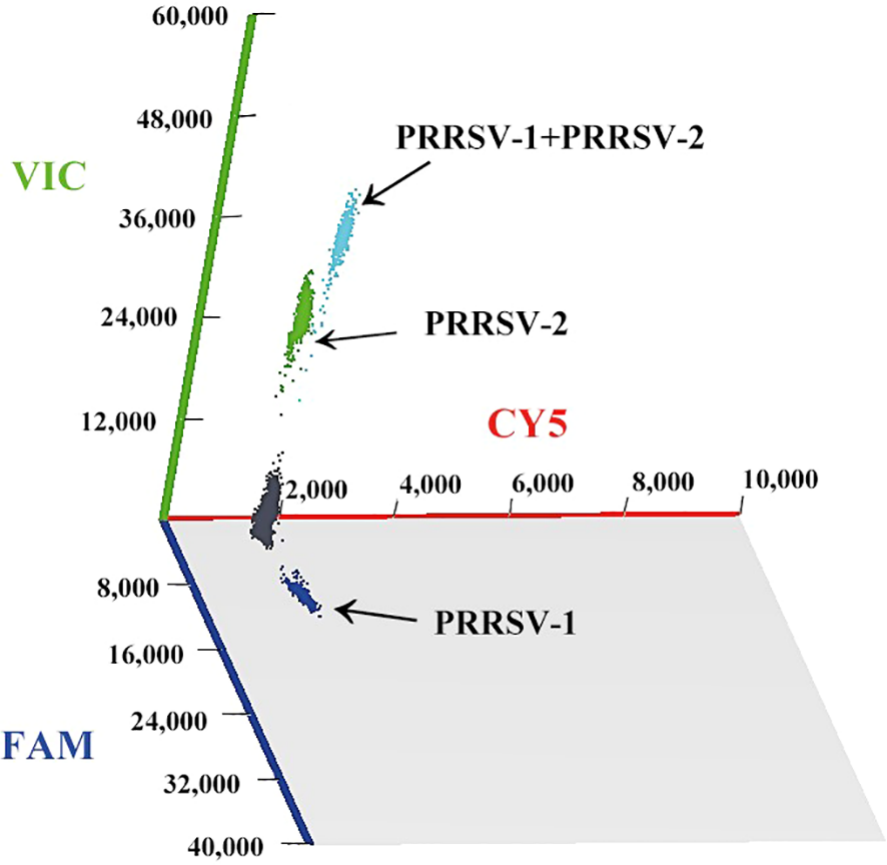
The 3D scatter plots of fluorescence intensity of the clinical samples. The fluorescence intensities of PRRSV–1 and PRRSV–2 were collected in the FAM and VIC acquisition channels, respectively.

From April 2024 to May 2025, the 2,185 clinical samples were collected, and tested by the established duplex cdPCR. The results showed that the positivity rates of PRRSV–1 and PRRSV–2 were 2.20% (48/2,185) and 23.43% (512/2,185), respectively, and the co–infection rate of PRRSV–1 and PRRSV–2 was 1.05% (23/2,185) (**Table 5**). The positive samples were displayed in 3D dot–plots, and the data was directly visualized via a three–dimensional scatter plots (**Figure 8**).

**Table 5:** Test results of the duplex cdPCR for the clinical samples in different time.

Time	Number	Number of positive samples		
		PRRSV–1	PRRSV–2	PRRSV–1+PRRSV–2
Apr, 2024	297	3	39	2
May, 2024	95	2	18	0
Jun, 2024	105	0	13	0
Jul, 2024	74	0	18	0
Aug, 2024	134	1	15	0
Sep, 2024	108	1	14	0
Oct, 2024	204	23	79	14
Nov, 2024	59	0	22	0
Dec, 2024	214	2	62	1
Jan, 2025	59	7	18	0
Feb, 2025	79	6	57	4
Mar, 2025	504	3	111	2
Apr, 2025	137	0	27	0
May, 2025	116	0	19	0
Total	2,185	48	512	23
Positivity rate (%)		2.20	23.43	1.05

The samples from pig farms, slaughterhouses, and harmless treatment plants showed the positivity rates of 5.09% (14/275), 1.85% (19/1,028), 1.70% (15/882) for PRRSV–1, and of 36.36% (100/275), 18.97% (195/1,028), 24.60% (217/882) for PRRSV–2 (**Table 6**).

**Table 6:** Test results of the duplex cdPCR for the clinical samples from different sources.

Location	Number	Number of positive samples		
		PRRSV–1	PRRSV–2	PRRSV–1+PRRSV–2
Pig farm	275	14 (5.07%)	100 (36.36%)	5 (1.81%)
Slaughterhouse	1,028	19 (1.85%)	195 (18.97%)	13 (1.26%)
Harmless treatment plant	882	15 (1.70%)	217 (24.60%)	5 (5.69%)
Total	2,185	48 (2.20%)	512 (23.43%)	23 (1.05%)

The 2,185 clinical samples were also tested by the WOAH-recommended duplex qPCR and the established duplex qPCR in this study to evaluate the clinical application of the established duplex cdPCR. Compared with the WOAH-recommended duplex qPCR, the clinical sensitivity and specificity of the duplex cdPCR for PRRSV–1 were 100% and 99.72%, and for PRRSV–2 were 100% and 99.64% (**Table 7**). Compared with the duplex qPCR, the clinical sensitivity and specificity of the duplex cdPCR for PRRSV–1 were 100% and 99.70%, and for PRRSV–2 were 100% and 99.77% (**Table 7**). The coincidence rate of the three methods was above 99.73% (**Table 7**).

**Table 7:** Clinical sensitivity and specificity of the developed duplex cdPCR from comparing with the duplex qPCR, and the WOAH-recommended qPCR.

Pathogen	Sample	Positive			Negative			Clinical Sensitivity (95% CI)		Clinical Specificity (95% CI)		Consistency rate	
		cdPCR	qPCR	W–qPCR	cdPCR	qPCR	W–qPCR	qPCR	W–qPCR	qPCR	W–qPCR	qPCR	W–qPCR
PRRSV–1	2,185	48	44	42	2,137	2,141	2,143	100.00%	100.00%	99.81%	99.72%	99.82%	99.73%
PRRSV–2	2,185	512	507	506	1,673	1,678	1,679	100.00%	100.00%	99.70%	99.64%	99.77%	99.73%

cdPCR refers to the developed duplex cdPCR; qPCR refers to the developed duplex qPCR; W–qPCR refers to the WOAH–recommended qPCR.

A total of 50 positive samples were selected randomly for ORF5 and Nsp2 gene amplification and sequence analysis to confirm the detection results of the duplex cdPCR. The obtained sequences have been uploaded to the NCBI GenBank, and will be analyzed in another paper (unpublished data).

## 4 Discussion

Digital PCR does not rely on standard curves and has high degree of specificity and sensitivity, thus it is applied in multiple fields, such as medicine, environment, food, and other industries (Gutiérrez–Aguirre et al., 2015; Kuypers and Jerome, 2017; Li et al., 2018; Brara et al., 2025; Gleerup et al., 2025; Koziaeva et al., 2025; Michel et al., 2025). This technology is also applied in the field of veterinary clinical practice. Yang et al. designed specific primers and probes for the PRRSV ORF7 gene and developed a dPCR, which was 10 times more sensitive than that of the qPCR (Yang et al., 2017). Long et al. established a dPCR based on the PRRSV Nsp2 gene to simultaneously detect and distinguish classical PPRSV, HP–PRRSV and NADC30–like PRRSV (Long et al., 2023). Shi et al. established a multiplex cdPCR for the differential detection of ASFV (p72 gene), CSFV (5' untranslated region), and PRRSV (ORF7 gene) (Shi et al., 2022). Shi et al. developed a triplex cdPCR to detect and distinguish porcine respiratory coronavirus (PRCV), PRRSV, and SIV (Shi et al., 2025). Even if PRRSV–1 and PRRSV–2 are still widespread in pig herds at present, no cdPCR has been reported for their differential detection. Therefore, a novel duplex cdPCR has been developed and validated in this study.

In this study, two pairs of specific primers and probes capable of distinguishing PRRSV–1 and PRRSV–2 were designed based on the conserved region of the ORF6 gene. The PRRSV strains used for multiple sequence alignments came from different countries around the world in different years, to ensure the specificity for PRRSV species and the universality of different PRRSV epidemic strains of the designed primers and probes. After optimizing the reaction conditions and procedures, the standard curves were fabricated. The R^2^ values were all above 0.998, indicating a good linear relationship. The assay was able to specifically detect only PRRSV–1 and PRRSV–2. The LODs of the duplex qPCR were 134.129 copies/reaction and 137.897 copies/reaction for PRRSV–1 and PRRSV–2, respectively, and the LODs of the duplex cdPCR were 4.507 copies/reaction and 5.607 copies/reaction for PRRSV–1 and PRRSV–2, respectively, indicating that the duplex cdPCR was 30 times (for PRRSV–1) and 25 times (for PRRSV–2) more sensitive than the established duplex qPCR, respectively. The sensitivity of the developed duplex cdPCR was similar to those of Long et al. (8 copies/reaction) (Long et al., 2023) and Shi et al. (6 copies/reaction) (Shi et al., 2022), and more sensitive than those of Yang et al. (38 copies/reaction) (Yang et al., 2017) and Shi et al. (12 copies/reaction) (Shi et al., 2025). In the previous reports, the LODs of the duplex qPCR for the differential detection of PRRSV-1 and PRRSV-2 established by Ruan et al. (2023) (LODs of 160 copies/reaction for PRRSV-1, and 94 copies/reaction for PRRSV-2), Chae et al. (2023) (LODs of 20 copies/reaction for PRRSV-1 and PRRSV-2), Ren et al. (2025) (LODs of 200 copies/reaction for PRRSV-1, and 20 copies/reaction for PRRSV-2), and Tian X. et al. (2025) (LODs of 16 copies/reaction for PRRSV-1 and PRRSV-2) were higher than that of the developed duplex cdPCR in this study, which indicated that the developed assay was suitable for testing the clinical samples with very low viral load. The developed assay had good repeatability, with less than 2% CVs for the intra– and inter–assay. The clinical samples were detected by three methods: the established duplex cdPCR, the established duplex qPCR, and the reference WOAH-recommended qPCR. The positivity rates of PRRSV–1 were 2.20%, 2.01%, and 1.92%, while those of PRRSV–2 were 23.43%, 23.20%, and 23.16%, respectively. The clinical sensitivity and specificity of the duplex cdPCR were higher than 99.64%, with the consistency rates exceeding 99.73%. The results indicated that the positivity rates of the duplex cdPCR was higher than those of the other two methods, suggesting that the duplex cdPCR was the most sensitive and accurate method for detecting PRRSV–1 and PRRSV–2 in the clinical samples. The results that the cdPCR is more sensitive than the corresponding qPCR further confirmed the results of the previous reports (Shi et al., 2022; Long et al., 2023; Shi et al., 2024). These data confirmed the accuracy and reliability of the developed duplex cdPCR. Even if these samples were collected from Guangxi province in South China, the genetic analysis of PRRSV strains in the previous reports showed that the PRRSV strains from Guangxi province belonged to PRRSV–1 and PRRSV–2 (Lineage 1, 3, 5, and 8), and were similar to the strains from other provinces in China [Hong et al., 2019; Wang et al., 2020, Wang et al., 2022]. This excluded the geographical and genetic limitations of the clinical samples from Guangxi Province and confirmed all validation data derive from samples collected in Guangxi Province. All these results indicate that a duplex cdPCR has been successfully established to detect and distinguish PRRSV–1 and PRRSV–2 simultaneously. Of course, the clinically prevalent PRRSV strains include PRRSV–1 and PRRSV–2, and PRRSV–2 further includes classical PRRSV, HP-PRRSV, NADC30-like, NADC34-like, QQYZ-like strains, etc, showing high diversity of PRRSV epidemic strains. In addition, PRRSV is a highly variable RNA virus that is constantly mutating, showing high diversity of genetic characteristics. These might lead to primer mismatches and missed detection of clinically positive samples due to PRRSV variability. These situations require us to continuously conduct clinical monitoring, be aware of the genetic diversity of the current epidemic strains, and design specific primers and probes for these strains, in order to timely and accurately detect them.

Of the detection results of the clinical samples, the positivity rates of PRRSV–1 and PRRSV–2 were 2.20%, and 23.43%, respectively. This indicated that PRRSV–2 was the dominant strain circulating in pig herds, which has been detected in 14 cities of Guangxi Province. On the contrary, the positivity rate of PRRSV–1 was relatively low, and found only in a few cities in Guangxi Province. Since 1996, PRRSV–2 has been popular widespread in pig herds throughout China, and the pig industry has been suffered huge economic losses (Guo et al., 2018; Zhou et al., 2024). Therefore, a lot of effective measures has been proposed and implemented for prevention and control of PRRSV–2 (Guo et al., 2018; Han et al., 2017; He et al., 2025). However, the positivity rate of PRRSV–1 has been relatively low since its discovery in China (Huang et al., 2024; Li et al., 2024; Huang et al., 2024). The clinical samples from different provinces in China during 1994–2024 showed that the PRRSV–1 positivity rate ranged from 0.26% to 32%, whereas most reports showed less than 3% positivity rate for PRRSV–1 (Huang et al., 2024). The 1,618 clinical samples from Sichuan province in China during 2021–2023 showed a prevalence rate of 39.74% (643/1,618) for PRRSV, of which PRRSV–1 was 4.35% (28/643), and PRRSV–2 was 95.65% (615/643) (Huang et al., 2024). The clinical samples from five provinces in China during 2020–2021 showed 17.54%–53.33% of PRRSV–positive individuals, and of the positive samples for sequence analysis, 14 samples (2.74%) were PRRSV–1 and 497 samples (97.26%) were PRRSV–2 (Li et al., 2024). This study showed that PRRSV–1 had positivity rate of 2.20% in Guangxi Province. However, pigs infected with PRRSV–1 usually also show clinical signs such as high fever and dyspnea, and pathological changes such as interstitial pneumonia, which are similar to those of PRRSV–2 and difficult to distinguish (Lunney et al., 2010; Weesendorp et al., 2014; Sánchez-Carvajal et al., 2021; Ruedas-Torres et al., 2024). The severity of the disease and the losses of PRRSV–1 to the pig industry has been far underestimated in China (Sun et al., 2023; Huang et al., 2024). Even if PRRSV–2 is still the dominant genotype, the detection rate of PRRSV–1 has been increasing in recent years (Huang et al., 2024; Li et al., 2024; Huang et al., 2024). We should strengthen the detection and epidemiological investigation of this disease in order to grasp the true situation of its occurrence and prevalence, and propose targeted prevention and control measures to minimize its harm to the pig industry. The developed duplex cdPCR provides useful method for sensitive and accurate detection of PRRSV–1 and PRRSV–2, which is the first step towards accurate diagnosis and timely intervention of the disease. This method is especially suitable for the case with low concentration of viral templates, and timely measures could be taken in the early stage of swine disease to reduce economic losses.

It is noteworthy that PRRSV–1 and PRRSV–2 were co-circulating in the clinical cases, with co–infection rate of 1.05% in this study. According to the previous reports, the phenomenon of co–infection with PRRSV–1 and PRRSV–2 is common in pig herds. It was reported that after the live PRRSV vaccines were used in Denmark, the PRRSV–1 and PRRSV–2 have coexisted in pig herds (Oleksiewicz et al., 1998). The clinical samples from five provinces in China between 2019 and 2024 showed the co–infection rate of 1.69% (6/356) for PRRSV–1 and PRRSV–2 (Tian X. et al., 2025). From 2010 to 2011, Thailand conducted a survey of 102 pig farms, and PRRSV–1 and PRRSV–2 were presented in 100 pig herds (Nilubol et al., 2013). From 2018 to 2020, 1,847 positive samples were found in 5,062 samples from South Korean farms, and 6% (113/1,847) samples were coinfected with PRRSV–1 and PRRSV–2 (Lee et al., 2023). During 2019 to 2020, the 15,034 serum or tissues samples from Taiwan Province of China showed 36.34% (5,464/15,034) positivity rate of PRRSV, of which 0.21% (31/15,034) were co–infected with PRRSV–1 and PRRSV–2 (Hsueh et al., 2023). These results indicated that PRRSV–1 and PRRSV–2 co-infection is an important phenomenon, which has to be considered while effective measures are taken for prevention and control of PRRS.

Recombination and variation usually occur in PRRSV–1 and PRRSV–2 (Sun et al., 2017; Eclercy et al., 2019; Sun et al., 2022; Yu et al., 2022; Tu et al., 2024; Wang et al., 2024; Gao et al., 2025; Tian Z. et al., 2025). Different strain recombination of PRRSV occurred between wild–wild strains, vaccine–wild strains, as well as between vaccine–vaccine strains (Eclercy et al., 2019; Sun et al., 2022; Gao et al., 2025). The virulence of the recombinant strain is enhanced, and recombination also leads to a decrease of the effectiveness of the vaccine, which aggravates the seriousness of the disease to pigs (Sun et al., 2017; Tu et al., 2024; Tian Z. et al., 2025). The recombination also increases the difficulty to epidemiological surveillance (Yu et al., 2022; Wang et al., 2024). Therefore, the conservative and variant regions of the targeted gene sequences have to be considered while designing the specific primers and probes for the development of qPCR and dPCR. In this study, the gene sequences of the PRRSV ORF6 gene were obtained from NCBI GenBank (**Supplementary Table S1**), and multiple sequence alignments were done. The conserved regions were selected for designing the specific primers and probes, which ensured the specificity of the developed duplex cdPCR.

## 5 Conclusions

This study successfully established a duplex cdPCR that can simultaneously detect and distinguish PRRSV–1 and PRRSV–2 for the first time. This method has good sensitivity, specificity, and repeatability, and provides a reliable tool for the differential detection of PRRSV–1 and PRRSV–2 in the clinical samples. The test results of the clinical samples confirm that PRRSV–2 strains are still the predominant genotype circulating in pig herds in South China, but the prevalence of PRRSV–1 cannot be ignored.

## Data Availability

The original contributions presented in the study are included in the article/**Supplementary Material**. Further inquiries can be directed to the corresponding authors.
