# Supplementary material for: Development of a novel duplex crystal digital PCR for the detection of PRRSV–1 and PRRSV–2
Source: Front Cell Infect Microbiol. 2026 Feb 27;16:1701517. doi: 10.3389/fcimb.2026.1701517 (PMC12982388; doi:10.3389/fcimb.2026.1701517)
Supplement: Supplementary file 1 [file Table1.docx]

**Supplementary Materials**

**Table S1** The PRRSV reference strains used in this study.

| **Strain** | **Accession Number** | **Source** | **Year** | **Genotype** |
| --- | --- | --- | --- | --- |
| 9625/2012 | KJ415276.1 | Hungary | 2012 | 1 |
| Amervac PRRS | GU067771.1 | Spain | Vaccine | 1 |
| AUT13-883 | KT326148.1 | Austria | 2013 | 1 |
| Cresa3256 | JF276432.1 | Spain | 2005 | 1 |
| GZ11-G1 | KF001144.1 | China | 2011 | 1 |
| PUST-2789-3W-2 | MN242825.1 | China | 2018 | 1 |
| Olot/91 | KF203132.1 | Spain | 1991 | 1 |
| P073-3 | MK214314.1 | China | 2015 | 1 |
| SHE | GQ461593.1 | China | 2009 | 1 |
| BJEU06-1 | GU047344.1 | China | 2006 | 1 |
| FJEU13 | KP860912.1 | China | 2013 | 1 |
| HeB3 | MN927227.1 | China | 2018 | 1 |
| HENZMD-10 | KY363382.1 | China | 2016 | 1 |
| HL85 | MN927229.1 | China | 2018 | 1 |
| HLJB1 | KT224385.1 | China | 2014 | 1 |
| KZ2018 | MN550991.1 | China | 2018 | 1 |
| LNEU12 | KM196101.1 | China | 2012 | 1 |
| NVDC-MN11-2011 | JX187609.1 | China | 2011 | 1 |
| SC-2020-1 | MW115431.1 | China | 2020 | 1 |
| TZJ236 | OP566682.1 | China | 2020 | 1 |
| HK3 | KF287129.1 | China | 2003 | 1 |
| HKEU16 | EU076704.1 | China | 2007 | 1 |
| DK-2003-7-2 | KC862572.1 | Denmark | 2003 | 1 |
| NMEU09-1 | GU047345.1 | China | 2009 | 1 |
| NVDC-NM2 | KC492504.1 | China | 2011 | 1 |
| 13V117 | KT159249.1 | Belgium | 2013 | 1 |
| Lelystad virus | M96262.2 | Netherlands | 1991 | 1 |
| PRRS-FR-2005-29-24-1 | KY366411.1 | France | 2005 | 1 |
| CHsx1401 | KP861625.1 | China | 2014 | 2 |
| FJ1402 | KX169191.1 | China | 2014 | 2 |
| FJZ03 | KP860909.1 | China | 2013 | 2 |
| HNjZ15 | KT945017.1 | China | 2015 | 2 |
| IA/2014/NADC34 | MF326985.1 | USA | 2014 | 2 |
| JL580 | KR706343.1 | China | 2015 | 2 |
| MN184A | DQ176019.1 | USA | 2001 | 2 |
| HNhx | KX766379.1 | China | 2016 | 2 |
| NADC30 | MH500776.1 | USA | 2008 | 2 |
| FJFS | KP998476.1 | China | 2012 | 2 |
| GM2 | JN662424.1 | China | 2011 | 2 |
| HNyc15 | KT945018.1 | China | 2015 | 2 |
| QYYZ | JQ308798.1 | China | 2011 | 2 |
| BJ-4 | AF331831.1 | China | 1996 | 2 |
| HN1 | AY457635.1 | China | 2003 | 2 |
| RespPRRS MLV | AF066183.4 | USA | 1994 | 2 |
| VR-2332 | AY150564.1 | USA | 2003 | 2 |
| CH-1a | AY032626.1 | China | 1996 | 2 |
| CH-1R | EU807840.1 | China | 2008 | 2 |
| GX1003 | JX912249.1 | China | 2010 | 2 |
| GZgy17 | MK144542.1 | China | 2017 | 2 |
| HeNan-A9 | KJ546412.1 | China | 2013 | 2 |
| HH08 | JX679179.1 | China | 2011 | 2 |
| HUN4 | EF635006.1 | China | 2006 | 2 |
| JA142 | AY424271.1 | USA | 1997 | 2 |
| JXA1 | EF112445.1 | China | 2006 | 2 |
| JXAI P80 | FJ548853.1 | China | 2008 | 2 |
| JXwn06 | EF641008.1 | China | 2009 | 2 |
| P129 | AF494042.1 | USA | 2002 | 2 |
| SCya18 | MK144543.1 | China | 2018 | 2 |
| TJ | EU860248.1 | China | 2006 | 2 |
| TJbd14-1 | KP742986.1 | China | 2014 | 2 |
| R98 | DQ355796.1 | China | 2006 | 2 |
